# Supplementary material for: Mechanisms governing the pioneering and redistribution capabilities of the non-classical pioneer PU.1
Source: Nat Commun. 2020 Jan 21;11:402. doi: 10.1038/s41467-019-13960-2 (PMC6972792; doi:10.1038/s41467-019-13960-2)
Supplement: Supplementary file 7 — Source data [file 41467_2019_13960_MOESM7_ESM.zip › Source_Data/Figure6/Figure6B_MotifScanOutput/FLI1_PU.1/homerResults/motif24.similar.html]

motif24

## Information for motif24

C
A
T
G
A
C
T
G
A
T
C
G
A
T
C
G
A
T
C
G
T
C
A
G
A
C
T
G
  
Reverse Opposite:  

T
G
A
C
A
G
T
C
A
T
G
C
T
A
G
C
T
A
G
C
T
G
A
C
G
T
A
C
  

|  |  |
| --- | --- |
| p-value: | 1e-53 |
| log p-value: | -1.221e+02 |
| Information Content per bp: | 1.682 |
| Number of Target Sequences with motif | 2436.0 |
| Percentage of Target Sequences with motif | 24.48% |
| Number of Background Sequences with motif | 6382.9 |
| Percentage of Background Sequences with motif | 17.49% |
| Average Position of motif in Targets | 100.0 +/- 56.2bp |
| Average Position of motif in Background | 98.1 +/- 69.9bp |
| Strand Bias (log2 ratio + to - strand density) | -0.0 |
| Multiplicity (# of sites on avg that occur together) | 1.41 |
| Motif File: | file (matrix) reverse opposite |

### Similar de novo motifs found

|  |  |  |  |  |  |  |  |
| --- | --- | --- | --- | --- | --- | --- | --- |
| Rank | Match Score | Redundant Motif | P-value | log P-value | % of Targets | % of Background | Motif file |
| 1 | 0.806 | A C T G A C T G A C T G A G T C A C T G A C T G C T A G C T G A | 1e-45 | -104.231067 | 15.29% | 10.06% | motif file (matrix) |
| 2 | 0.764 | A C T G A C T G A G T C A C T G A C T G A C T G C T A G A G T C | 1e-37 | -85.604067 | 9.55% | 5.79% | motif file (matrix) |
